# Glucose Metabolism, Lactate, Lactylation and Alzheimer’s Disease

**DOI:** 10.14336/AD.2025.0338

**Published:** 2026-06-20

**Authors:** Shuangshuang Hai, Yadan Hou, Meiyan Zhang, Xiaoyan Gao, Tuo Yang, Xiuli Shang, Xiaohong Sun

**Affiliations:** ^1^Department of Geriatrics, The Fourth Affiliated Hospital of China Medical University, Shenyang, Liaoning 110032, China.; ^2^Department of Neurology, Chifeng Municipal Hospital, Chifeng, Inner Mongolia 024000, China.; ^3^Department of Neurology, The First Affiliated Hospital of China Medical University, Shenyang, Liaoning 110000, China.; ^4^Department of Neurology, Central Hospital of Dalian University of Technology, Dalian, Liaoning 116033, China.; ^5^Science Experiment Center, China Medical University, Shenyang, Liaoning 110122, China.

**Keywords:** Alzheimer's disease, lactate, lactylation, tau, neuroinflammation

## Abstract

Alzheimer's disease (AD) is a neurodegenerative disorder primarily characterized by cognitive decline; however, its pathogenesis remains incompletely understood. In recent years, the role of lactate metabolism and its derived lactylation modifications in AD has received increasing attention. As a product of glycolysis, lactate is not only a key molecule in energy metabolism but also regulates gene expression and protein function through lactylation modifications. Studies have shown that in the brains of AD patients, glucose metabolism is significantly reduced, while glycolysis is upregulated, and lactate levels are elevated. Nevertheless, the research regarding the relationship between lactylation and AD remains limited. Building on recent advances in understanding lactylation in neurodegenerative diseases and related conditions, we analyze and explore the potential relationships between lactylation and AD from the perspectives of β-amyloid (Aβ) deposition, tau protein pathology, and neuroinflammation. In summary, lactylation, as a novel post-translational modification, holds significant promise in elucidating the pathological mechanisms and advancing the treatment of AD. A deeper investigation into its molecular mechanisms and regulatory networks may open new avenues for the diagnosis and treatment of AD.

## 1. Introduction

Alzheimer's disease (AD), the most common form of dementia, is characterized by progressive cognitive decline [1]. With global population aging, AD has emerged as a significant public health challenge. Epidemiological data indicate that approximately one-third of individuals aged over 85 are affected by AD [2,3], and approximately 10% of individuals aged ≥65 years develop the disease [4]. By 2050, the number of AD patients is projected to increase from the current 50 million to 150 million [5]. AD progression is clinically categorized into preclinical, mild cognitive impairment (MCI), and dementia stages [2]. Early symptoms primarily include memory loss, language dysfunction, and other cognitive deficits. As the disease progresses, behavioral disturbances, motor impairment, and a decline in independent daily living skills may occur [6]. AD pathogenesis involves a combination of non-modifiable factors (age, apolipoprotein E ε4 (APOE ε4) genotype) and modifiable factors (hypertension, diabetes, obesity) [7-10]. Pathologically, AD is characterized by the deposition of extracellular β-amyloid (Aβ) plaque, intracellular tau neurofibrillary tangles, and chronic neuroinflammation [11,12]. The abnormal deposition of Aβ outside neurons and the hyperphosphorylation of tau protein inside neurons are the main pathological features of AD [13]. The overproduction or insufficient clearance of Aβ leads to its accumulation in the brain, forming amyloid plaques and causing neuronal toxicity. The hyperphosphorylated tau protein leads to the formation of neurofibrillary tangles, which disrupt neuronal structure and function, ultimately resulting in cell death [14]. Moreover, the abnormal activation of microglia and chronic inflammatory responses further exacerbate neuronal damage [15,16]. Current diagnostics rely on neuropsychological testing and biomarkers (Aβ/tau levels in cerebrospinal fluid, PET imaging), while treatments—cholinesterase inhibitors, N-methyl-D-aspartate (NMDA) receptor antagonists, and newer anti-Aβ/τ antibodies—provide limited symptomatic relief and encounter practical implementation challenges [17-19].

Although the brain comprises only 2-3% of body weight, it accounts for over 20% of the body’s energy [20-22]. Mounting evidence links metabolic dysfunction—particularly due to impaired glucose utilization and mitochondrial deficits—to AD. In AD neurons, glucose uptake and oxidative phosphorylation decline, and glycolysis is upregulated to compensate, resulting in elevated lactate accumulation [23]. Previously regarded as a mere waste product, lactate is now recognized as an energy substrate, signaling molecule, and epigenetic regulator through lysine lactylation [24]. Studies indicate that lactate levels in the cerebrospinal fluid of patients with AD are associated with tau protein pathology and cognitive decline, suggesting that lactate metabolism dysregulation may play a significant role in disease progression [25]. Dysregulated lactate metabolism and aberrant lactylation may exacerbate neuroinflammation and oxidative stress, pointing to potential novel therapeutic targets [26]. The present review systematically summarizes recent advances in our understanding of lactate metabolism and lactylation in AD and discusses potential interventions targeting these pathways.

## 2. Glucose metabolism in the brain and its disruption in AD

**Figure 1.**
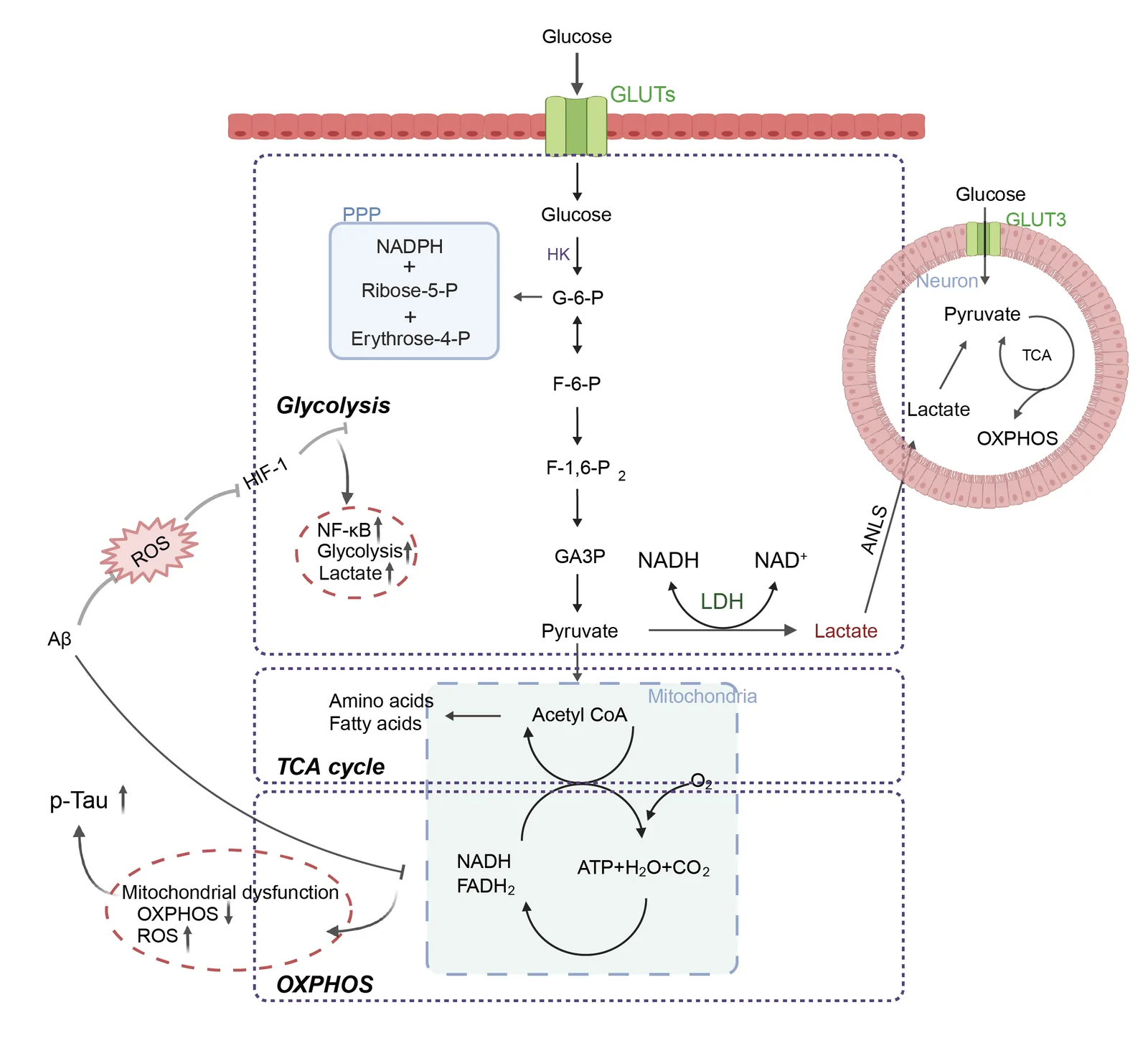
**Schematic Diagram of Brain Glucose and Lactate Metabolism**. Glucose crosses the blood-brain barrier (BBB) into the brain via glucose transporters (GLUT) and is phosphorylated by hexokinase (HK) to produce glucose-6-phosphate (G-6-P). G-6-P can initially undergo glycolysis, resulting in the production of two pyruvate molecules, which can then enter the mitochondria for metabolism through the tricarboxylic acid (TCA) cycle and oxidative phosphorylation (OXPHOS). Pyruvate can be reduced to lactate-by-lactate dehydrogenase (LDH). This lactate can be released into the extracellular space via monocarboxylate transporters (MCT). Additionally, G-6-P can generate NADPH through the pentose phosphate pathway (PPP). Dysfunction of glucose metabolism in the brain is a critical factor in the development of Alzheimer's disease (AD). Glucose is converted to pyruvate through glycolytic enzymes in astrocytes. Under hypoxic conditions, pyruvate is catalyzed to lactate by LDHA, which can then be transported to the astrocyte-neuron interstitial space via MCTs, subsequently taken up by neurons through MCT2. Lactate is converted back to pyruvate by LDH, and pyruvate enters the TCA cycle to produce energy, meeting the needs for synaptic transmission and neuronal excitability. In AD, Aβ reduces the efficiency of oxidative phosphorylation in mitochondria, leading to increased levels of reactive oxygen species (ROS), stabilizing HIF-1α, and inducing genes responsive to HIF-1, including those involved in glycolysis. This ultimately results in upregulation of glycolysis, mitochondrial dysfunction, neuroinflammation, and tau pathology in the brains of individuals with AD. Abbreviations: F-6-P, fructose-6-phosphate; F-1,6-P2, fructose-1,6-bisphosphate; GA3P, glyceraldehyde-3-phosphate; ANLS, astrocyte-neuron lactate shuttle; LDH, lactate dehydrogenase.

Brain energy metabolism represents a complex interplay among glucose metabolism, lactate metabolism, and various signaling pathways that are essential for maintaining neural function. Glucose metabolism primarily occurs through three interconnected pathways: glycolysis, the tricarboxylic acid (TCA) cycle, and oxidative phosphorylation (OXPHOS) (Fig. 1) [27]. Initially, glucose undergoes glycolysis in the cytoplasm, producing two pyruvate molecules, two ATP molecules, and two nicotinamide adenine dinucleotides (NADH) [28]. Under aerobic conditions, pyruvate is transported into the mitochondria where it is converted into acetyl-CoA by pyruvate dehydrogenase (PDH). This acetyl-CoA then enters the TCA cycle, where it is fully oxidized to CO_2_ while generating NADH and flavin adenine dinucleotide (FADH_2_) [22,29]. These reducing equivalents supply electrons to the electron transport chain (ETC), composed of four enzyme complexes (I-IV) that transfer these electrons to oxygen, ultimately forming water [22,30]. This electron transport drives proton pumps that generate a transmembrane proton gradient, facilitating ATP synthesis via ATP synthase—ultimately yielding approximately 36 ATP molecules per glucose molecule [31]. As cells with high energy demands, neurons primarily rely on mitochondrial OXPHOS for energy to support synaptic transmission, ion pump function, and cellular homeostasis. Recent insights suggest that neuronal somata may engage more in aerobic glycolysis compared to axonal terminals under both resting and activated conditions [30].

In AD, glucose metabolism is significantly impaired even before the onset of clinical symptoms [23]. The initial metabolic alterations include reduced glucose uptake, increased glycolysis, and mitochondrial dysfunction [23]. Glucose transporters (GLUTs) are key transmembrane proteins that facilitate glucose entry into cells [32]. GLUT1, primarily located in the endothelial cells of the blood-brain barrier (BBB) and astrocytes, as well as GLUT3, predominantly found in neurons, are critical for glucose transport [33,34]. In AD, the expression level of GLUT1 and GLUT3 is significantly reduced, thereby limiting the energy supply to neurons [35]. Interestingly, while insulin enhances the activity of GLUT4 in peripheral tissues, GLUT1 and GLUT3 are regulated by nutrient availability and metabolic conditions within the brain. Positron emission tomography (PET) imaging has demonstrated that abnormal glucose metabolism in AD exhibits regional specificity, notably in the temporoparietal lobe and posterior cingulate cortex, which correlates with cognitive decline [36,37].

The reduction in glucose metabolism in these regions is closely linked to cognitive decline. Enhanced glycolysis emerges as a characteristic metabolic response in AD, compensating for mitochondrial dysfunction while also contributing to disease progression [38]. Increased mitochondrial fission results in fragmentation and reduced OXPHOS capacity, which further diminishing ATP production [39]. In response, neurons upregulate key glycolytic enzymes such as hexokinase (HK) and lactate dehydrogenase, indicating an increase in glycolysis activity [40]. Furthermore, the activation of hypoxia-inducible factor-1α (HIF-1α) in AD promotes glycolysis [41] and enhances GLUT1 and GLUT3 expression, thereby supporting this metabolic shift [42]. This aberrant glycolytic pathway is associated with shifts in the NADH/NAD^+^ ratio, resulting in increased production of reactive oxygen species (ROS), which intensifies oxidative damage in neurons [43]. Altered metabolite levels, including lactate and ROS, may also drive pathological processes involving Aβ and tau proteins, thus exacerbating neurodegeneration [40]. Throughout these metabolic shifts, AD transitions from dependence on efficient mitochondrial OXPHOS to reliance on less efficient glycolysis, failing to meet the neuroenergetic demands and fostering an environment conducive to oxidative damage and increased pathology.

## 3. Lactate metabolism in the brain

### 3.1 Lactate production and function

Lactate, primarily existing as lactate ions (Lactate^-^) under physiological conditions, is produced by the reduction of pyruvate catalyzed by lactate dehydrogenase (LDH) [44]. Traditionally viewed as a byproduct of anaerobic metabolism, lactate also serves as a significant energy source and signaling molecules in the brain. Under hypoxic conditions, glycolysis is accelerated, resulting in increased lactate production that sustains ATP supply. Even in the presence of sufficient oxygen, cells such as astrocytes and highly active neurons can generate lactate through "aerobic glycolysis," often referred to as the Warburg effect [45].

In response to increased energy demands during heightened neural activity—such as learning and memory—neurons require a rapid supply of ATP, which leads to the activation of glycolytic pathway. Astrocytes, in turn, respond through calcium signaling to convert glucose into lactate, which is subsequently released into the extracellular space via monocarboxylate transporters (MCT) 4[46]. This initiates the astrocyte-neuron lactate shuttle (ANLS) [47], where neurons uptake lactate via high-affinity MCT2, converting it back into pyruvate for OXPHOS [48].

Lactate also facilitates communication between the brain and muscles during physical activity, serving as an "energy currency" [49]. Furthermore, its production fluctuates according to circadian rhythms, with higher lactate levels during wakefulness compared to sleep. During fasting or periods of glucose scarcity, lactate oxidation becomes essential for supplying energy to neurons [50]. These processes exemplify the brain's adaptive capacity to maintain energy balance through efficient metabolism of lactate.

### 3.2 Consequences of lactate metabolism imbalance

An imbalance in lactate metabolism primarily arises from dysregulated metabolic homeostasis, affecting the coordination of key energy metabolism pathways. Excessive activation of glycolytic enzymes (such as hexokinase and phosphofructokinase) due to AMPK or HIF-1α signaling causes glycolytic flux to surpass physiological thresholds, leading to excess lactate production [50,51]. Concurrently, impaired mitochondrial OXPHOS, due to enzyme dysfunction or increased ROS exacerbates the "Warburg effect," leading to a heightened reliance on glycolysis. Dysregulation of lactate transporters, including overexpression of MCT1 and MCT4 along with inhibition of MCT2, disrupts the lactate gradient across cell membranes, resulting in intracellular lactate accumulation [52,53]. Hormonal imbalances, such as insulin resistance and inflammatory cytokines, drive cells toward glycolytic dominance by affecting glucose transporter function and activating pathways like NF-κB.

This multifaceted imbalance leads to a failure of the feedback loop governing lactate production and clearance. Lactate, which signifies an energy crisis, also contributes to microenvironmental acidification that can inhibit enzyme activity and activate apoptotic pathways, perpetuating a cycle of dysfunction. In neurodegenerative diseases, as well as in cerebral ischemia and cancer, metabolic dysregulation is often linked with interactions between mitochondria and endoplasmic reticulum, as well as epigenetic changes, making it a crucial factor in disease progression [54,55].

### 3.3 Lactate metabolism in AD

Cerebrospinal fluid (CSF) lactate levels have emerged as potential biomarkers of the severity and progression in neurodegenerative disorders, reflecting both impaired energy metabolism and neuroinflammation [56,57]. Several studies suggest that AD patients have elevated CSF lactate levels compared to healthy controls, although the research is limited and findings are inconsistent. Elevated lactate levels may also correlate with inflammatory processes in the early stages of AD [58].

Investigations have shown that in early AD stages, CSF lactate levels can rise alongside decreased cerebral glucose metabolism, particularly in brain regions linked to the default mode network (DMN) [59]. Magnetic resonance spectroscopy (MRS) studies indicate that AD patients exhibit higher glucose, lactate, and ascorbate levels, with myo-inositol levels correlating with AD biomarkers like p-tau181P and total tau protein. This indicates a metabolic shift towards increased local glycolytic activity, which is closely related to cognitive decline [60].

In AD mouse models, acute hyperglycemia can elevate interstitial levels of Aβ and lactate by activating ATP-sensitive potassium channels, implicating abnormalities in glucose metabolism related to Aβ production [61]. Furthermore, individuals with diabetes are at an increased risk of developing AD, highlighting the connection between metabolic dysfunction and neurodegeneration [62]. Accumulated Aβ oligomers can inhibit the entry of mitochondrial pyruvate, disrupting the TCA cycle, while lactate-induced acidosis may activate glycogen synthase kinase-3β (GSK-3β), thereby promoting tau hyperphosphorylation.

Although upregulated glycolysis may initiallly compensate for neuronal energy deficits, it leads to lactate accumulation and futher neuronal dysfunction. Preclinical studies show that interventions aimd at reducing glycolytic flux or restoring mitochondrial biogenetics can effectively lower celebral lactate levels and improve cognitive performance in AD models [63]. Hence, dysregulated lactate metabolism represents a key aspect of energy dysfunction in AD, potentially driving neurodegenerative processes through micro-environmental acidosis and oxidative stress. These observations underscore lactate's integral role in the brain metabolism and highlight its pathways as promising targets for therapeutic modulation AD.

## 4. Lactylation

Lactylation is a recently recognized post-translational modification (PTM) characterized by the covalent attachment of lactate to lysine residues on proteins. This modification creates lactylation marks that regulate a variety of biological functions, including gene expression, metabolic reprogramming, and cell signaling [26]. Initially characterized on histone H3, lactylation has since been observed on a wide array of proteins, thus enhancing lactate's role from being merely a metabolic byproduct to a critical signaling molecule [26,64].

### 4.1 Regulatory mechanisms

The concept of the "lactate clock" provides insights into the timing and regulation of lactylation [26]. This modification is driven by elevated levels of lactate and the activity of specific enzymes. Lactylation can be either enzymatic, catalyzed by p300 using lactyl-CoA as a substrate, or non-enzymatic, resulting from direct interactions between lactate and lysine residues under conditions of high lactate concentration [65,66]. Key factors influencing lactylation include lactate accumulation, often triggered by enhanced glycolysis or mitochondrial dysfunction, as well as the balance between lactylation enzymes and their regulators. Pathological conditions such as hypoxia, inflammation, and metabolic changes further impact this process. Lactylation alters protein charges and conformations, thereby influencing their activity, stability, and interactions, and plays a significant role in mechanisms such as inflammation, angiogenesis, and fibrosis [67,68].

Moreover, lactylation can enhance the function of DNA repair proteins such as meiotic recombination 11 homolog (MRE11) and Nijmegen breakage syndrome 1 (NBS1) by promoting processes that facilitate DNA damage repair [69,70]. However, in AD, the combined effects of oxidative stress, mitochondrial dysfunction, and Aβ toxicity often lead to a rate of DNA damage accumulation that far exceeds the compensatory capacity of the repair systems, resulting in a net accumulation of DNA lesions [71]. Current research suggests that lactylation primarily enhances nuclear DNA repair efficiency, while mitochondrial DNA (mtDNA) remains particularly vulnerable to oxidative damage due to its lack of protective histones [72,73]. Furthermore, lactylation may preferentially upregulate homologous recombination repair pathways, providing insufficient compensation for critical processes such as base excision repair [74]. Chronic inflammation and Aβ toxicity in AD may establish a pathological positive feedback loop—where pathological lactate accumulation not only drives the modification of repair proteins but also depletes NAD+, induces tau hyperphosphorylation, and exacerbates neuroinflammation, ultimately creating a vicious cycle of "enhanced repair capacity yet diminished fidelity [28,75].

Furthermore, lactylation influences immune responses by enhancing the release of high-mobility group box 1 (HMGB1) in macrophages, thereby promoting inflammation [76]. It also stimulates angiogenesis through the upregulation of factors such as leucine-rich alpha-2 glycoprotein 1 (LRG1) and vascular endothelial growth factor (VEGF) [77,78]. These interactions underscore the multifaceted roles of lactate in cellular processes and disease.

### 4.2 Crosstalk with other PTMs

Lactylation exhibits complex interactions with other PTMs, including acetylation, methylation, and phosphorylation, which jointly regulate protein functions and signaling pathways. This crosstalk involves competition at modification sites and influences enzyme activity and cellular metabolic states [79]. In the tumor microenvironment, lactic acid promotes the lactylation of histone H3 lysine 18 (H3K18), inhibiting acetylation-dependent gene expression and reshaping the immunosuppressive milieu [80].

The enzymes involved in lactylation often share overlapping functionalities. For instance, the acetyltransferase p300/CBP can catalyze both acetylation and lactylation, while histone deacetylases (HDACs) and the sirtuin family exhibit dual specificity; however, their efficiency in removing lactylation is significantly lower than that for deacetylation [69,81,82]. In the context of macrophage polarization, acetylation predominates in the early activation of pro-inflammatory genes, while lactylation facilitates the promotes the conversion to an anti-inflammatory phenotype at later stages by stabilizing heterochromatin structures [26].

An imbalance between lactylation and acetylation under pathological conditions can drive disease progression. In gliomas, the synergistic action of lactylation and acetylation modulation activates pathways such as Wnt/β-catenin, contributing to radioresistance [83]. In lung cancer models, lactylation of cancer-associated fibroblasts (CAFs) enhances the EGFR/Akt signaling cascade and IL-6 expression, further amplified by NF-κB signaling. This cascading effect illustrates how metabolic modifications can act as upstream regulators within classical signaling networks.

Lactylation also impacts gene expression through its interactions with methylation. For instance, lactylation at histone H3K18 affects chromatin accessibility, influencing the occurrence and impact of methylation modifications. The interplay between lactylation and methylation may significantly affect transcriptional activities, particularly in cancer and neurodegenerative diseases, presenting new therapeutic targets [84].

In conclusion, lactylation represents a critical PTM that expands our understanding of cellular signaling and metabolic regulation, particularly in the context of disease. Its complex interactions with other PTMs highlight its significance as a regulatory node in various biological processes, suggesting potential avenues for therapeutic intervention in metabolic and neurodegenerative disorders.

## 5. Lactylation in AD

### 5.1 Tau protein

Tau is a microtubule-associated protein predominantly expressed in the central nervous system, characterized by three primary domains: the assembly, projection, and proline-rich domains [85]. In the human brain, tau exists in six isoforms generated by alternative mRNA splicing, distingguished by the presence or absence of the second repeat sequence and variations in N-terminal inserts [86]. Tau stabilizes microtubules, thus maintaining axonal integrity and facilitating intracellular transport within neurons [87]. In AD, abnormal modifications and aggregation of tau are recognized as core pathological features. Key biomarkers for early diagnosis include phosphorylated tau (p-tau) levels, particularly p-tau181 and p-tau217, which are found in CSF and blood [88].

Hyperphosphorylation leads to tau detachment from microtubules and subsequent aggregatation into neurofibrillary tangles (NFTs), which destabilize microtubules and impair neuronal transport and synaptic function, ultimately contributing to cognitive decline. In addition to phosphorylation, other abnormal modifications such as acetylation, ubiquitination, SUMOylation, and truncation, further exacerbate tau dysfunction and neuronal degeneration [89,90].

Lactylation alters tau protein by attaching lactate to specific lysine residues, which affects its charge and spatial conformation. These changes increase tau’s susceptibility to protease recognition and cleavage, particularly by δ-secretase [91]. Lactylation enhances the accessibility of cleavage sites within insoluble tau fractions, promoting proteolytic cleavage. Molecular dynamics simulations have revealed that lactylation induces structural changes in tau, significantly increasing the flexibility of specific residues that may facilitate protease activity. Additionally, lactylation inhibits ubiquitination-mediated degradation, thereby prolonging tau's half-life and increasing its cleavage likelihood.

Lactylation significantly influences tau hyperphosphorylation, particularly at K331 site. Lactate treatment has been shown to increase tau lactylation and promoting both its phosphorylation and cleavage. Inhibition of lactate production, for example, through knocking down lactate dehydrogenase A(LDHA), results in decreased tau lactylation. The acetyltransferase p300 catalyzes tau lactylation at the K331 site by interacting with it as lactyl-CoA [91]. In AD, downregulation of isocitrate dehydrogenase 3β (IDH3β) results in mitochondrial dysfunction and lactate accumulation, which futher enhances tau hyper-phosphorylation, contributing to cognitive symptoms. The reduction of IDH3β leads to OXPHOS uncoupling, decreased energy metabolism, and lactate accumulation. Lactate, acting as a donor of lactyl groups, promotes histone lactylation, particularly at histone H4 lysine 12 (H4K12la) and histone H3 lysine 18 (H3K18la), thereby enhancing the expression of paired box gene 6 (PAX6). As an inhibitory transcription factor of IDH3β, PAX6 further suppresses IDH3β expression, forming a positive feedback loop of "IDH3β-lactate-PAX6-IDH3β"[92]. This cycle ultimately leads to tau hyperphosphorylation, synaptic damage, and impairments in learning and memory. In 5xFAD mice, upregulating IDH3β or downregulating PAX6 improves energy metabolism, reduces histone lactylation, and significantly alleviates AD-like pathologies, including tau hyperphosphorylation, synaptic damage, and memory deficits [93]. Therefore, lactylation may exacerbate tau pathological aggregation and neurotoxicity by promoting phosphorylation.

Tau ubiquitination, primarily mediated by E3 ubiquitin ligases such as CHIP, is crucial for its degradation and the preventing accumulation [94]. Experimental evidence indicates that lactylation inhibits tau polyubiquitination, suggesting a potential mechanism by which lactylation stabilizes tau [91,95]. Conversely, knocking down LDHA or employing the tau3KR mutant (which limits tau lactylation) enhanced tau ubiquitination, supporting the notion that lactylation interferes with normal degradation pathways [91].

### 5.2 Amyloid protein

Amyloid Precursor Protein (APP) is a transmembrane protein widely expressed in various tissues, particularly abundant in neurons. APP consists of multiple domains, including a large extracellular domain, a transmembrane domain, and a short intracellular domain [96]. Its extracellular domain contains several functional regions, such as the Kunitz-type protease inhibitor (KPI) domain and metal ion-binding sites, which are involved in cell-cell interactions and signal transduction [97]. Under physiological conditions, APP is processed through two main pathways: the non-amyloidogenic pathway and the amyloidogenic pathway [98]. In the non-amyloidogenic pathway, APP is cleaved by α-secretase, generating soluble APP fragments (sAPPα), which have neuroprotective properties and synaptic plasticity regulatory functions [99]. In the amyloidogenic pathway, APP is sequentially cleaved by β-secretase (BACE1) and γ-secretase, resulting in the production of Aβ[100]. Aβ exists in two main forms: Aβ40 and Aβ42, with Aβ42 considered a key molecule in AD pathology due to its stronger aggregation propensity and neurotoxicity [101,102]. Structurally, Aβ42 consists of 42 amino acids, with its N-terminus exposed externally and a hydrophobic core (composed predominantly of hydrophobic amino acids) located internally [103]. This structure enables Aβ42 to readily form oligomers and fibrils, which subsequently deposit as amyloid plaques [104]. The formation of amyloid plaques is recognized as one of the primary pathological features of AD, inducing neuronal damage and neuroinflammation, ultimately leading to cognitive decline. The physiological functions of Aβ are not fully understood, but studies suggest that it may participate in neuronal signaling and synaptic plasticity regulation under normal conditions [105]. However, overproduction of Aβ or impaired clearance mechanisms can lead to its abnormal aggregation, triggering neurotoxicity characterized by oxidative stress, mitochondrial dysfunction, and neuronal apoptosis [106]. Additionally, Aβ activates microglia and astrocytes, exacerbating neuroinflammation.

Lactylation may reduce Aβ production by modulating the metabolic pathway of APP. In APP/PS1 double-transgenic mice, lactylation at the K612 site of APP alters its interaction with BACE1, thereby reducing the cleavage of APP by BACE1 that generates Aβ fragments. Furthermore, lactylation promotes the interaction between APP and CD2AP, accelerating the degradation of APP through the endosomal-lysosomal pathway and further limiting Aβ accumulation [107]. This finding suggests a potential therapeutic role of lactylation in AD. The neurotoxicity of Aβ is closely related to its oligomeric forms and oxidative stress. Lactylation may mitigate or exacerbate the pathological effects of Aβ through two pathways: antioxidant and anti-inflammatory actions, as well as the regulation of mitochondrial function [24,108]. On one hand, lactylation may reduce the accumulation of ROS by regulating the expression of antioxidant genes such as superoxide dismutase (SOD) and glutathione prexidase (GPx), thereby inhibiting Aβ-induced oxidative damage. Additionally, lactylation might suppress the pro-inflammatory response of microglia, mitigating neuroinflammation associated with Aβ deposition and indirectly lowering its toxicity. On the other hand, lactylation activates PINK1-mediated mitophagy, clearing damaged mitochondria and maintaining mitochondrial homeostasis, which in turn reduces Aβ-induced neuronal apoptosis and synaptic dysfunction [109]. These mechanisms suggest that lactylation may play a significant neuroprotective role in regulating Aβ toxicity, while also potentially participating in the pathological process of AD through its effects on mitochondria function and inflammatory responses.

Although the existing literature does not directly address the interaction between lactylation and other PTMs in regulating Aβ, mechanisms elucidated for other known PTMs provide clues for understanding potential interactions. While the phosphorylation of Aβ itself has been less studied, it may indirectly regulate Aβ production by influencing the activity of related enzymes [107]. Lactylation may compete or synergize with phosphorylation at adjacent sites, thereby altering kinase and phosphatase activity, or influencing substrate conformation, ultimately affecting the balance of phosphorylation. For example, lactylation may modulate the activity of glycolysis-related enzymes, thereby altering ATP levels and subsequently influencing phosphorylation regulation [110]. Moreover, N-terminal pyroglutamate modification of Aβ enhances its oligomerization and neurotoxicity, a modification that depends on glutaminyl cyclase; lactylation may affect the efficiency of this modification by altering local pH or enzyme activity [111,112]. Additionally, lactylation may regulate protein stability or conformation, exposing or concealing sites of pyroglutamate modification, which could exacerbate Aβ pathological aggregation [113]. The ubiquitination system plays a critical role in clearing abnormal proteins; therefore, lactylation may interfere with the degradation efficiency of Aβ oligomers by affecting the activity or substrate recognition of ubiquitin ligases [114]. Furthermore, oxidative modifications are closely related to oxidative stress in AD [115]. As a metabolism-related modification, lactylation may accumulate during mitochondrial dysfunction and compete with oxidative modifications (e.g., nitration or carbonylation) for the same lysine residues [116]. This competition may alter the redox sensitivity of Aβ, potentially affecting its oligomer formation or binding affinity to metal ions such as copper or zinc [117].

### 5.3 Lactylation and neuroinflammation

Recent studies have revealed the critical role of neuroinflammation in the progression of AD. Neuroinflammation exhibits bidirectional regulatory relationships with Aβ and tau pathology and serves as a key mechanism driving neuronal damage and cognitive decline [75]. Postmortem and imaging studies confirm that the "amyloid cascade hypothesis" alone cannot fully explain the neurodegeneration in AD, while the chronic activation of microglia and astrocytes is central to the persistent inflammatory response in the AD brain [118].

Infiltration of peripheral immune cells, including T cells and monocytes, has been observed in the AD brain. These cells enter the brain parenchyma through BBB disruption and interact with local glial cells, creating a pro-inflammatory microenvironment. Interleukin-17(IL-17) produced by γδ T cells in the meninges has been shown to accelerate amyloid pathology and cognitive deficits, and neutralizing IL-17 significantly delays disease progression [119].

Neuroinflammation directly leads to neuronal death by activating the pyroptosis pathway. Studies have demonstrated gasdermin D (GSDMD)-mediated pyroptosis in the hippocampus of AD patients, involving multiple cell types, including microglia, astrocytes, and neurons [120]. Moreover, calcium homeostasis dysregulation drives metabolic shifts in microglia (from OXPHOS to glycolysis), forming a vicious cycle of inflammation-energy metabolism [121]. Additionally, animal experiments show that inhibiting tumor necrosis factor-α (TNF-α) simultaneously reduces Aβ deposition and tau pathology, highlighting inflammation as a shared regulatory node [122].

### 5.3.1 Microglia

Microglia, the resident immune cells of the central nervous system, detect Aβ deposition via pattern recognition receptors (e.g., TREM2, TLR) and initiate inflammatory responses [123]. This process involves the activation of the NLRP3 inflammasome, leading to the release of pro-inflammatory cytokines such as IL-1β and IL-18, which directly exacerbate neuronal death [124]. Notably, Aβ itself may exhibit cytokine-like functions, and its release can trigger innate immune cascades, thereby unifying the amyloid hypothesis and neuroinflammation theory [125].

Microglia in AD exhibit functional heterogeneity: they play a neuroprotective role by clearing Aβ in the early stages but transform into a pro-inflammatory phenotype upon chronic activation, secreting cytokines such as TNF-α and IL-6 to exacerbate synaptic loss [126]. Aβ can activate microglia by binding to Toll-like receptors (TLR4/6), and the IL-1β secreted by microglia further promotes the expression of β-secretase, increasing Aβ production [127]. Galectin-3 (Gal3) has been shown to promote the conversion of Aβ monomers into insoluble fibrils, forming an inflammation-driven amplification mechanism of amyloid pathology [128]. TREM2 agonist antibodies enhance microglial phagocytosis and clearance of Aβ while inhibiting their transition to a pro-inflammatory phenotype [129]. NLRP3 inhibitors (e.g., MCC950) have demonstrated the ability to simultaneously improve amyloid pathology and cognitive function [130]. In manganese-induced neurotoxicity models, microglial activation leads to elevated levels of pro-inflammatory cytokines and ROS, accompanied by the leakage of mtDNA into the cytoplasm, further activating the cGAS-STING signaling pathway and driving neuroinflammation and neuronal apoptosis [131]. Additionally, single-cell RNA sequencing has revealed the presence of disease-associated microglia (DAM) in AD, whose abnormal metabolic pathways may promote a chronic inflammatory state [132].

The pro-inflammatory activation process of microglia involves a metabolic shift from OXPHOS to glycolysis. Lactate influences the energy state and functional phenotype of microglia through metabolic reprogramming. In the brains of 5xFAD mice and individuals with AD, elevated levels of H4K12la have been observed in microglia adjacent to Aβ plaques, predominantly enriched in the promoter regions of glycolytic genes (such as HIF-1α, LDHA), activating their transcription and thereby increasing glycolytic activity [133]. This regulation forms a positive feedback loop: H4K12la upregulates the expression of pyruvate kinase M2 (PKM2), a key enzyme in glycolysis, which further promotes lactate production, driving additional H4K12la modifications [133]. This positive feedback mechanism exacerbates glycolytic activity and metabolic dysregulation in the brains of AD patients. This metabolic shift not only reduces the energy supply to neurons but also increases lactate accumulation, further impairing neuronal function. Pharmacological inhibition of PKM2 weakens microglial activation, while microglial-specific deletion of PKM2 improves spatial learning and memory in AD mice [133]. Disrupting this positive feedback loop may represent a potential therapeutic strategy for AD.

Cellular senescence is closely related to AD. One study showed significantly elevated lactate levels in senescent microglia and hippocampal tissue of AD model mice (FAD 4T and APP/PS1), which increased the levels of global histone Kla. H3K18la and Pan-Kla were significantly upregulated in senescent microglia and hippocampal tissue of AD model mice. Enhanced H3K18la directly stimulates the NFκB signaling pathway by increasing its binding to the promoters of Rela (p65) and NFκB1 (p50), thereby upregulating senescence-associated secretory phenotype (SASP) components like IL-6 and IL-8[134]. Under hypoxic conditions, lactate increases in microglia, leading to the lactylation of the non-histone protein Yin Yang-1 (YY1) at K183, which is regulated by p300. Over-lactylated YY1 directly enhances the transcription of fibroblast growth factor 2(FGF2) and promotes angiogenesis [68]. Additionally, YY1 lactylation contributes to promoting the activation of microglia, enhancing their proliferation and migration capabilities [135]. H4K12la is significantly elevated in microglia after spinal cord injury (SCI). Lactate treatment can promote the proliferation, scar formation, axon regeneration, and restoration of motor function in microglia following SCI. The specific mechanism may be related to lactate-mediated elevation of H4K12la promoting the transcription of PD-1 in microglia. These results illuminate the roles and mechanisms of the lactate/H4K12la/PD-1 signaling pathway in microglia-mediated tissue repair [136]. Exercise is considered a promising non-pharmacological intervention to improve the pathology of neurodegenerative diseases. Research has found that running exercise can ameliorate the hyperactivation of microglia, increase their anti-inflammatory/reparative phenotype, and enhance cognitive function. This process involves an increase in lactate in the body. Elevated lactate can act as a "promoter" of an endogenous "lactate timer" in microglia, facilitating the transformation of microglial phenotypes through lactylation [137].

Cyclic GMP-AMP synthase (cGAS) activates the STING signaling pathway in microglia by sensing cytoplasmic DNA (such as mDNA or pathogen DNA), thereby driving the formation of a DAM phenotype. This phenotype is closely linked to neuroinflammation, synaptic damage, and the pathology of AD [138]. Inhibition of cGAS can reduce the accumulation of microglia around amyloid plaques and protect synaptic integrity and neuronal function. AD risk factors such as APOE ε4 and TREM2 R47H enhance cGAS-STING signaling, inducing microglial senescence and neurotoxic phenotypes, suggesting that cGAS is a key molecule connecting genetic risk with neurodegeneration [139]. In AD models, the upregulation of the cGAS-STING pathway in microglia promotes the release of pro-inflammatory factors and activates the NLRP3 inflammasome, exacerbating neuroinflammation; it also regulates the expression of IFITM3 (interferon-induced transmembrane protein 3) to promote M1 polarization, worsening Aβ-induced neuroinflammation [140,141]. Lactylation can inhibit cGAS-mediated innate immune signaling both *in vitro* and *in vivo*, hindering the recognition and activation of DNA by cGAS and directly suppressing its enzymatic activity and IFN-I production [142]. These results demonstrate the dual role of lactylation in AD [143].

### 5.3.2 Astrocytes

Astrocytes are the most abundant glial cells in the central nervous system. They are responsible for maintaining the metabolic homeostasis of neurons, including regulating synaptic function and the integrity of the BBB [144,145]. Astrocytes exhibit functional abnormalities early in AD. Single-cell sequencing studies have identified disease-associated astrocytes (DAAs) in both AD mouse models and human patients. These cells appear early in the disease and progressively increase in number as the disease progresses [146]. DAAs show altered gene expression profiles, with dysregulation in pathways related to neuroinflammation, metabolic disturbances, and metal ion homeostasis, suggesting that they may promote AD pathogenesis by modulating the microenvironment [147].

Astrocytes initially clear Aβ plaques through phagocytosis. However, in AD, their metabolic functions are compromised. Aβ treatment can lead to metabolic dysregulation of urea in astrocytes, resulting in the production of excessive neurotoxic metabolites (such as nitrites), which, in turn, damages neurons and impairs memory function [148]. Moreover, the APOE subtype secreted by astrocytes can promote Aβ deposition and affect synaptic clearance through interaction with microglia [149].

In AD, astrocytes transition to a reactive phenotype, releasing inflammatory factors and inhibitory neurotransmitters. For instance, reactive astrocytes excessively synthesize gamma amino butyric acid (GABA) through the monoamine oxidase B (MAOB) pathway, inhibiting neuronal activity and leading to memory deficits [150]. Additionally, chitinase-3-like protein 1 (CHI3L1), a marker of astrocyte activation, is elevated in the CSF of AD patients and may exacerbate neuronal damage by mediating the association between Aβ and tau pathology [151]. In the preclinical stage of AD, astrocyte activation may promote the spread of tau pathology via inflammatory signals [152]. In animal models, inhibiting the astrocyte-specific gene BAG3 (B-cell lymphoma 2-associated athanogene 3) can reduce the accumulation of tau and α-synuclein, suggesting a direct role in the pathological deposition of proteins [153]. In astrocytes from AD patients and P301S tau transgenic (PS19) mice, the level of histone deacetylase 7 (HDAC7) is significantly increased. The upregulation of HDAC7 induces AD-like tau pathology by deacetylating the transcription factor EB (TFEB) and inhibiting lysosomal biogenesis in astrocytes, indicating that downregulating the HDAC7-TFEB signaling pathway may hold therapeutic potential for preventing AD [154].

Regarding Aβ metabolism, astrocytes directly control Aβ production through γ-secretase activity. For example, in the transgenic mouse model with early amyloid deposition, the expression of IFITM3 in astrocytes is increased. IFITM3 associates with γ-secretase and upregulates its activity, enhancing the production of Aβ. In late-onset AD patient samples, the content of IFITM3 in the γ-secretase complex is strongly positively correlated with γ-secretase activity [155]. In the regulation of tau pathology and neuroinflammation, the activation of astrocytes is associated with the abnormal phosphorylation of tau protein. Reactive astrocytes may promote initial tau phosphorylation through the release of unknown signaling molecules in individuals who are Aβ-positive but without significant tau pathology, thus linking Aβ deposition with subsequent neurofibrillary tangle formation. Overall, astrocytes may exhibit multifunctionality in AD and interact with various pathological features.

Under pathological conditions, such as SCI, cerebral ischemia, or subarachnoid hemorrhage, astrocytes enhance glycolytic activity to produce large amounts of lactate to meet energy demands. The high-lactate levels may regulate key metabolic enzymes in astrocytes (such as HK2) through lactylation modifications, like its role in preeclampsia, thereby altering cell proliferation and energy metabolism balance [156]. This represents a key mechanism linking oxidative stress with metabolic abnormalities. In ischemic stroke, the accumulation of lactate in the brain during the ischemic phase exacerbates brain injury by promoting the formation of Kla sites, leading to neuronal death and activation of A1-type astrocytes. Inhibition of LDHA or glycolytic pathways significantly mitigates brain damage, while additional lactate supplementation worsens the injury. Experimental evidence indicates that protein Kla primarily occurs in neurons, and astrocyte-derived lactate is a key driving factor. Specific knockout of LDHA in astrocytes or the use of the p300 inhibitor A-485 to block protein Kla formation significantly reduces the volume of brain infarction and improves neurological recovery [157].

Research shows that in astrocytes, low-density lipoprotein receptor-related protein 1 (LRP1) inhibits glucose uptake, glycolysis, and lactate production, resulting in decreased lactylation of ADP-ribosylation factor 1 (ARF1), which enhances the release and transfer of healthy mitochondria to neurons and alleviates ischemia-reperfusion injury in mouse models of ischemic stroke [115]. Furthermore, human studies confirm that CSF lactate levels in stroke patients are higher than those in normal controls, and CSF lactate levels show a positive correlation with infarction area and a negative correlation with mitochondrial levels in astrocytes. Following, single-cell sequencing data reveal an upregulation of the key glycolytic enzyme 6-Phosphofructo-2-kinase (PFKFB3) in astrocytes, promoting lactate production and further driving histone lactylation [158]. This forms a glycolysis-lactylation positive feedback loop that regulates inflammatory responses and regenerative repair. In the context of subarachnoid hemorrhage (SAH), astrocytes exhibit significantly enhanced glycolytic activity, leading to increased lactate production to sustain the energy requirements of neurons and other brain cells. After SAH, targeting the bromodomain protein 4 (BRD4) in astrocytes significantly reduces H4K8la, exacerbating the A1 polarization of astrocytes and ultimately impacting the recovery of neurological function and prognosis in mice [159]. In summary, lactate and lactylation modifications may profoundly influence the functions of astrocytes in both physiological and pathological conditions through metabolic regulation, activation of inflammatory signaling pathways, and intercellular interactions.

## 6. Modulating protein lactylation may be a potential intervention strategy for AD

**Figure 2.**
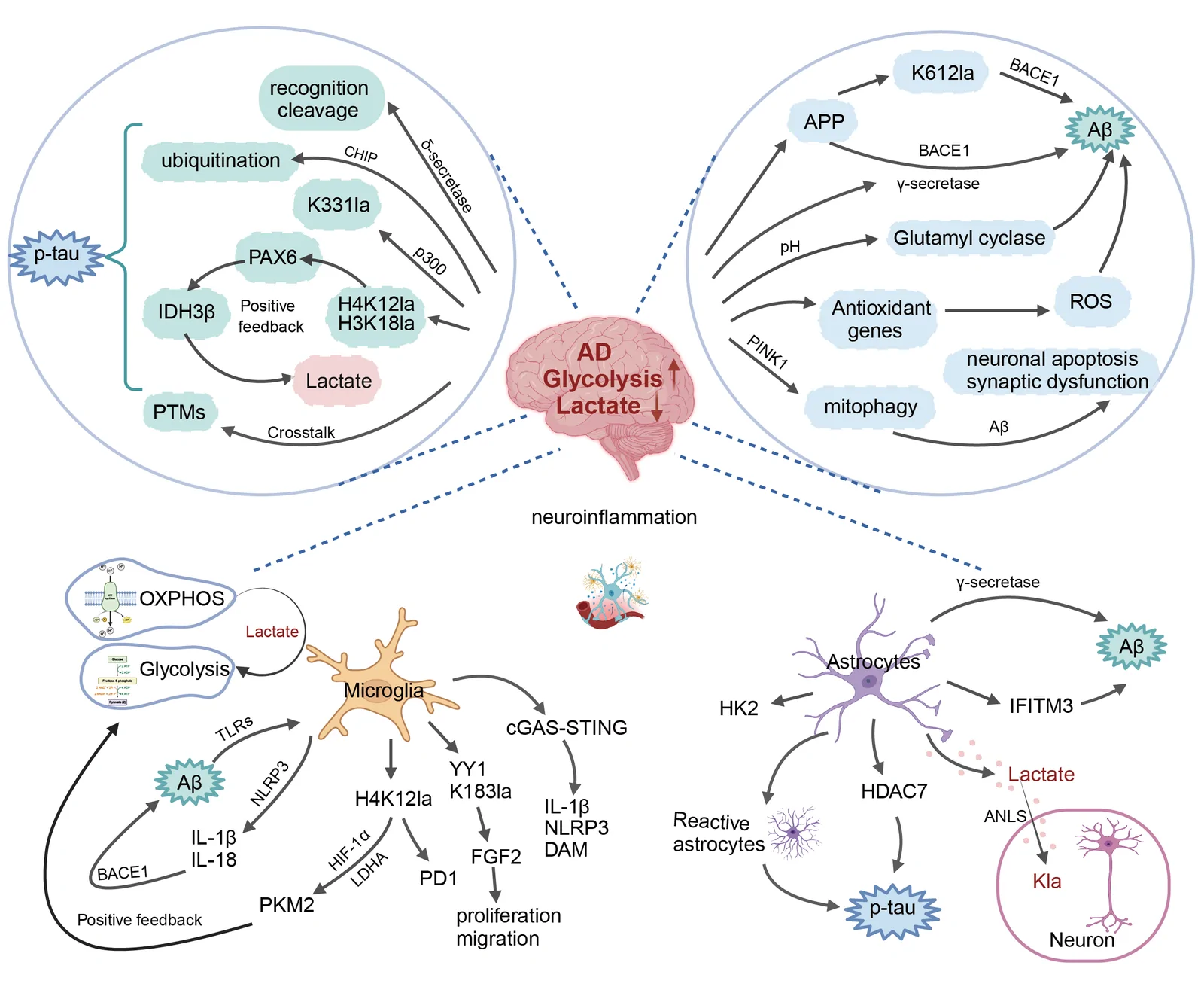
**Research progress on the interaction mechanisms of tau protein, amyloid protein, neuroinflammation, and lactylation**. Glucose is converted into lactic acid through the glycolytic pathway, and lactic acid is transformed into lactoyl-CoA, leading to lactylation of proteins via enzymatic or non-enzymatic pathways, which in turn affect their functions. Generated using Biorender. AD, Alzheimer's disease; CHIP, HSP70-interacting protein; IDH3β, isocitrate dehydrogenase 3β; PTMs, post-translation modifications; PAX6, paired box gene 6; APP, amyloid precursor protein; Aβ, amyloid-beta; BACE1, β-secretase; ROS, reactive oxygen species; OXPHOS, oxidative phosphorylation; TLRs, Toll-like receptors; PKM2, pyruvate kinase M2; YY1, Yin Yang-1; FGF2, fibroblast growth factor 2; DAM, disease-associated microglia; HK2, hexokinase 2; HDAC7, histone deacetylase 7; IFITM3, interferon-induced transmembrane protein 3; ANLS, astrocyte-neuron lactate shuttle.

Lactylation is widely present in brain tissue, particularly enriched in neurons and glial cells, suggesting its involvement in the pathological processes of AD through the regulation of neuronal excitability, synaptic plasticity, and energy metabolism (Fig. 2). In the brains of AD patients, general disturbance in glucose metabolism manifests as reduced glucose uptake, decreased glycolytic activity, and mitochondrial dysfunction. This metabolic reprogramming leads to increased lactate production or impaired clearance, subsequently affecting the levels of lactylation. In models of diabetes-associated cognitive decline (DACD), hyperglycemia-induced lactate accumulation significantly increases H4K12la and exacerbates mitochondrial oxidative stress by activating the FOXO1/PGC-1α signaling pathway, ultimately resulting in neuronal damage [160]. A similar mechanism may also exist in AD, considering the high overlap of pathological features related to insulin resistance and metabolic dysregulation between AD and diabetes. Enhancing lactate metabolism might counteract the cerebral glucose hypometabolism seen in AD, but its long-term effects and impact on neuronal health remain unclear.

Research on lactylation in AD has made significant progress, involving various aspects like amyloid protein metabolism, tau pathology, neuroinflammation, and metabolic dysregulation. Studies have found that amyloid precursor protein (APP) lactylation occurs at lysine 354 (APP-K354la), lysine 363 (APP-K363la), and lysine 612 (APP-K612la). Using lactylation mimic mutants, it was shown that APP-K612la, rather than the other two sites, significantly reduced C-terminal fragment-β (CTF-β) protein levels. Meanwhile, there were no changes in APP and its hydrolytic enzymes, including ADAM10, BACE1, and PS1, indicating that APP-K612la may influence APP transport and metabolism without affecting its expression. Researchers speculate that APPK612T may promote subsequent APP endocytosis by inhibiting α-secretase cleavage at the plasma membrane. The endocytosed lactylated APP might inhibit BACE1 binding and promote APP degradation via the endosome-lysosome pathway [107]. These findings suggest that targeting lactylation may offer a novel therapeutic strategy for AD; however, its efficacy in human patients remains unverified and warrants further investigation.

Furthermore, tau protein lactylation may regulate its phosphorylation, cleavage, and the formation of neurofibrillary tangles, while further exacerbating pathological damage by modulating ferroptosis and metabolic abnormalities [161]. Histone lactylation-promotes neuroinflammation and Aβ deposition by activating inflammasomes and inhibiting autophagy [162]. In macrophages, lactylation enhances the function of reparative macrophages, promoting inflammation resolution and tissue repair. This mechanism may extend to the activation of microglia in AD. Microglia in the brains of AD patients exhibit a pro-inflammatory phenotype (M1 type), and lactylation may inhibit neuroinflammatory responses by regulating the expression of inflammation-related genes. Notably, although inhibition of this pathway could ameliorate microglial dysfunction, excessive suppression may compromise microglial Aβ-clearance capacity.

Based on these findings, strategies targeting lactylation enzymes, regulating histone modifications, and reducing lactate production show therapeutic potential. Lactate dehydrogenase inhibitors, such as sodium pyruvate, can reduce lactate accumulation, thereby lowering the levels of lactylation. Additionally, pyruvate dehydrogenase kinase (PDK) inhibitors may promote the entry of pyruvate into the TCA cycle, reducing lactate production and improving neuronal energy metabolism [163]. Targeting the "writer" or "eraser" enzymes of lactylation could regulate the expression of AD-related genes. Specific inhibitors of lactylation, may exert neuroprotective effects by blocking the transcription of pro-inflammatory genes. Furthermore, targeting immunometabolic regulation also plays an important role. Animal studies have shown that TAK-242 (a TLR4 inhibitor) can enhance histone lactylation levels, promoting the differentiation of reparative macrophages (M2 type). Such strategies may also be applicable to the regulation of overactivated microglia in AD. Moreover, lactylation may affect the autophagic-lysosomal function by modifying key proteins in the autophagy pathway, such as mTOR or Beclin-1, thereby regulating the degradation of Aβ and tau [162]. These studies provide new insights into the pathological mechanisms and treatments for AD.

## 7. Summary and future directions

Lactylation, as a novel PTM, has seen significant breakthroughs in recent years across various fields, including cancer, metabolic disorders, and neurodegenerative diseases. Research has confirmed that this modification is widely present on lysine residues of both histone and non-histone proteins, influencing gene expression and protein function through a dynamic "writer-eraser-reader" regulatory system. In terms of metabolic regulation, lactylation acts as a "molecular sensor" for glycolytic flux, affecting energy metabolic homeostasis through modifications of metabolic enzymes like HK2. It has shown distinct modification patterns in pregnancy-related disorders such as preeclampsia [156]. In the nervous system, the level of lactylation in brain tissue is closely related to neuronal excitability, and lactate accumulation resulting from ischemic stroke may alter the modification patterns of synaptic proteins, impacting neurofunctional outcomes. Recent technological advancements, notably the application of genetic code expansion (GCE) techniques, have enabled the functional analysis of lactylated proteins at specific sites within live cells for the first time, providing new methods to precisely study the biological effects of these modifications [164]. With the integration of multi-omics analyses and artificial intelligence technologies, research on lactylation is transitioning from mechanistic exploration to clinical translation, particularly showing great promise in the development of inhibitors targeting lactylation enzymes (such as Pevonedistat) and optimizing immunotherapeutic strategies. This review systematically summarizes recent research progress on lactate and lactylation in AD. Given that direct studies on the relationship between AD and lactylation remain relatively scarce, we focus on the regulatory role of lactylation from three main aspects: the amyloid hypothesis, the tau hypothesis, and the neuroinflammation hypothesis. The discovery of lactylation not only opens new avenues for the study of PTMs but also offers a fresh perspective on the biological functions of lactate. Future research should concentrate on elucidating the differences in lactylation in neurons, astrocytes, and microglia in the brains of AD patients, identifying key target proteins, and exploring the time-dependent and dynamic roles of lactylation in regulating gene expression and cellular functions. This will help deepen our understanding of the mechanisms underlying cell-specific lactylation profiles. Additionally, it is essential to validate the dynamic changes of lactylated proteins in CSF or blood as potential biomarkers for AD. Moreover, interdisciplinary intervention strategies should be considered, such as metabolic regulation combined with epigenetic editing techniques, to explore multi-pathway synergistic intervention approaches that enhance treatment efficacy. It is important to note that lactylation is closely linked to glycolysis and energy metabolism; thus, targeting lactylation may interfere with other metabolic pathways and result in off-target effects, requiring careful management of the therapeutic window. Evaluating the effects of lactylation modulators on cognitive function and pathological markers using AD animal models and optimizing their BBB penetration, are critical steps for advancing clinical translation. These studies will provide important theoretical foundations and practical evidence for precision treatment of AD. In summary, the relationship between lactylation and AD warrants further exploration.
